# Genome mapping of postzygotic hybrid necrosis in an interspecific pear population

**DOI:** 10.1038/hortres.2015.64

**Published:** 2016-01-06

**Authors:** Sara Montanari, Lester Brewer, Robert Lamberts, Riccardo Velasco, Mickael Malnoy, Laure Perchepied, Philippe Guérif, Charles-Eric Durel, Vincent G M Bus, Susan E Gardiner, David Chagné

**Affiliations:** 1 Research and Innovation Centre, Fondazione Edmund Mach, Via Mach 1, 38010 San Michele all’Adige (TN), Italy; 2 The New Zealand Institute for Plant & Food Research Limited, Palmerston North Research Centre, Palmerston North, New Zealand; 3 Institut de Recherche en Horticulture et Semences - UMR1345, Institut National de la Recherche Agronomique (INRA), SFR 4207 Quasav, 42 rue Georges Morel, F-49071 Beaucouzé, France; 4 The New Zealand Institute for Plant & Food Research Limited, Motueka Research Centre, Motueka, New Zealand; 5 Institut de Recherche en Horticulture et Semences - UMR1345, Université d’Angers, F-49045 Angers, France; 6 The New Zealand Institute for Plant & Food Research Limited, Hawke’s Bay Research Centre, Havelock North, New Zealand.

## Abstract

Deleterious epistatic interactions in plant inter- and intraspecific hybrids can cause a phenomenon known as hybrid necrosis, characterized by a typical seedling phenotype whose main distinguishing features are dwarfism, tissue necrosis and in some cases lethality. Identification of the chromosome regions associated with this type of incompatibility is important not only to increase our understanding of the evolutionary diversification that led to speciation but also for breeding purposes. Development of molecular markers linked to the lethal genes will allow breeders to avoid incompatible inbred combinations that could affect the expression of important agronomic tratis co-segregating with these genes. Although hybrid necrosis has been reported in several plant taxa, including Rosaceae species, this phenomenon has not been described previously in pear. In the interspecific pear population resulting from a cross between PEAR3 (*Pyrus* *bretschneideri* × *Pyrus communis*) and ‘Moonglow’ (*P. communis*), we observed two types of hybrid necrosis, expressed at different stages of plant development. Using a combination of previously mapped and newly developed genetic markers, we identified three chromosome regions associated with these two types of lethality, which were genetically independent. One type resulted from a negative epistatic interaction between a locus on linkage group 5 (LG5) of PEAR3 and a locus on LG1 of ‘Moonglow’, while the second type was due to a gene that maps to LG2 of PEAR3 and which either acts alone or more probably interacts with another gene of unknown location inherited from ‘Moonglow’.

## Introduction

Hybrid necrosis (HN) is the reduced viability of a hybrid due to genetic incompatibilities. Although interactions between genes may have a positive effect on the hybrid, resulting in it having better performance than its parents (hybrid vigour), they may also be detrimental and cause sterility, weakness or lethality.^1^ Genetic incompatibilities can occur at different stages of the reproduction process, and they are generally divided into prezygotic and postzygotic, acting, respectively, before and after fertilization. HN, which is also termed hybrid weakness or inviability, is a class of postzygotic gene-flow barrier that is associated with a typical seedling phenotype, characterized by cell death, tissue necrosis, wilting, yellowing, chlorosis, dwarfism and reduced growth rate, and in some cases lethality.^2,3^ HN has been observed in several plant taxa, in wild and cultivated species and both in inbred populations and outcrosses; however, its phenotype appears to be characteristic across a range of hosts, suggesting a common underlying mechanism.^2,3^ According to the Bateson–Dobzhansky–Muller (BDM) model, the genetics of HN is simple and involves epistasis between at least two loci.^4^ The BDM model posits that independent substitutions occurring in two diverging lineages, not detrimental in their native genomic context, might be deleterious when combined in the hybrid. Most of the cases of HN reported in the literature are explained by a two-gene epistasis^2,5^; however, there are some examples of three-locus interactions^6^ and lethality controlled by a single locus.^7–9^

Although hybrid inviability has long been known amongst plant breeders and speciation scientists, with examples in the literature since the early 20th century,^7^ only recently have efforts been made to explain its molecular basis. The HN phenotype resembles the set of symptoms resulting from pathogen attack, and research on *Arabidopsis* spp.^1,6,10^ and tomato^11^ demonstrated that it was linked to autoimmunity reactions involving resistance (*R*) genes. During this hypersensitive response (HR), the plant undergoes oxidative stresses, followed by programmed cell death,^12,13^ in order to halt the spread of the pathogen, which requires living tissues.^14^ In the case of hybrid inviability, the plant immune system is improperly activated in the absence of a pathogen attack because of the genetic incompatibility, which causes tissue necrosis similar to that observed during HR. One hypothesis is that different (at least two) R proteins, encoded by independently evolved *R* genes, cause autonecrosis when they interact in the hybrid.^1^ Alternatively, one locus encodes a host protein, which regulates the activation of the R protein encoded by the second locus, as explained by the ‘guard hypothesis’.^3,15^ Most of the *R* genes demonstrated to be involved in HN belong to the Nucleotide Binding Leucine-Rich Repeats (NB-LRR) class. For example, Bomblies *et al*^1^ detected two unlinked regions (*DM1* and *DM2*) that were responsible for the HN in an *Arabidopsis thaliana* segregating population, and identified *DM1* as an NB-LRR gene. Moreover, they proved that genetic interaction between those two loci was required for increased resistance to *Hyaloperonospora parasitica*. When Alcázar *et al*^6^ investigated the cause of dwarfism in hybrids of two *A. thaliana* recombinant inbred lines, they found that Toll/Interleukin-1 Receptor-NB-LRR genes were the likely determinants of one of the interacting loci responsible for the phenomenon. This gene cluster mapped to the same position as the *DM2* locus detected by Bomblies *et al*.^1^ The work of Krüger *et al*^11^ in tomato (*Solanum lycopersicum* L.) was the first example of a ‘guard-guardee’ interaction causing genetic incompatibility. In *S. lycopersicum* lines introgressed with the *Cf-2* gene from a wild relative of tomato, *Solanum pimpinellifolium* Jusl., both autonecrosis and resistance to the fungus *Cladosporium fulvum* were observed. The two phenomena were dependent on the interaction between the *Cf-2* gene from *S. pimpinellifolium*, encoding for an LRR-containing receptor-like protein (the ‘guard’), and the *RCR3* locus from *S. lycopersicum*, encoding for a cysteine endoprotease (the ‘guardee’). However, when the *RCR3* locus was introduced from *S. pimpinellifolium*, no autonecrosis was observed, and the resistance was maintained. This demonstrated that the two loci were incompatible with each other only when they had evolved in different genomic contexts.

*R* genes, and especially LRR domains, are known to be highly polymorphic, even within the same species, evolving at very fast rates under the pressure of natural selection for resistance,^16^ and consistent with the hypothesis of their implication in BDM-like genetic incompatibilities. Indeed, there are several examples in the literature of HN events occurring in segregating populations developed to increase the resistance to pathogens in a range of species,^1,2^ including wheat,^17^ rice^18^ and diploid potatoes (*Solanum* spp.).^19^

Only a few examples of hybrid inviability have been reported for Rosaceae species. Loci linked to chlorotic or albino leaf, dwarfism and lethality have been detected in strawberry and apple. Sargent et al^20^ mapped a recessive locus for the pale-green leaf trait (*pg*) in the interspecific *Fragaria vesca* *F. nubicola* F2 progeny, which was suggested to be orthologous to the *vir* gene mapped in *Malus* spp. by Fernández-Fernández *et al*^21^ that was associated with the virescent phenotype in progeny from several East Malling rootstock crosses. In apple, a gene for compact habit was shown to be linked to the *Vf* gene for scab resistance,^22^ now called *Rvi6,*^23^ which maps to LG1. A few years later, Alston^24^ demonstrated that the pale-green lethal trait in apple, which characterizes seedlings deficient in chlorophyll that die a few weeks after germination, was controlled by the recessive gene *l*, linked to *Rvi6*. In addition, two different sublethal recessive genes (*sl1* and *sl2*), detected by Gao and Van de Weg^25^ in apple, were linked to the *Rvi6* gene. These genes control lethality at two different stages of apple seedling development, *sl1* after and *sl2* before germination, and they both interacted with another locus, *sl3*, whose map position was not identified. Distorted segregation ratios in favour or against scab resistance have been reported also in other publications, both in apple^26,27^ and in pear.^28,29^ Moreover, hybrid lethality has been described previously in intergeneric hybrids between apple and pear.^30,31^ More recently, Tsuruta and Mukai^32^ mapped a single locus (*HIs1*) associated with seedling inviability to LG4 of a cherry interspecific hybrid.

A pear interspecific segregating population was developed from a cross between PEAR3 (*Pyrus* *bretschneideri* × *Pyrus communis*) and ‘Moonglow’ (*P. communis*) at The New Zealand Institute for Plant & Food Research Limited (PFR Motueka, New Zealand), for the purpose of detecting chromosome regions linked to resistances against fire blight (*Erwinia amylovora*), pear scab (*Venturia pirina*) and pear psylla (*Cacopsylla pyri*).^33^ A subset of the seeds originating from this cross was planted and grown at PFR Motueka, and another subset at INRA, Angers (France). In both environments, stunted seedlings and lethality were observed and postulated to be due to HN. We describe the initial identification and subsequent validation of genomic regions linked to HN, using genetic mapping in populations consisting of both necrotic and non-necrotic plants.

## Materials and methods

### Plant material and growth conditions

Fruits were harvested from PEAR3 pollinated by ‘Moonglow’(cross described above) in Motueka during the summers (late February) of 2010 and 2014. Seeds were extracted, washed, treated with 10% Janola (42 g sodium hypochlorite/L) and dried, then stored in a refrigerator at 3–5°C until sowing. In winter 2010 (July), 760 seeds were sown in Motueka, with a further 728 sown in winter 2011 in Angers (February) and another 240 in winter 2014 in Motueka (July). In 2010 in Motueka, seeds were spread evenly through damp sphagnum moss for stratification, in order to break the dormancy, and stored in a refrigerator at 3–5°C until germination; then all seeds were planted. In Angers, seeds were also subjected to stratification in a moist sand and vermiculite substrate at 3–5°C for 3 months, after which they were planted in a mixture of peat and sphagnum soil. In 2014 in Motueka, seeds were dipped in 5% Thiram 40F (400 g L^−1^ Thiram as a suspension concentrate) before sowing to prevent fungal development, and then placed on filter paper into Petri dishes (Supplementary Figure 1), and 3 mL of 5% Thiram 40F was added to each plate. Petri dishes were sealed with Parafilm^®^ to prevent desiccation and stored at 3–5°C for 53 days, and then at 20°C for 3 days. On the second day at 20°C, they were again treated with Thiram 40F as above. Petri dishes were then moved back to 3–5°C until seed germination. Seeds were planted into pots containing Dalton’s strawberry potting mix 7 days after germination and moved to the greenhouse. The first batch of seeds was planted 67 days after extraction from the fruit and sowing continued on a weekly basis for another 75 days. During storage in the refrigerator, some seeds were treated a third time with Thiram 40F because of fungus development, while others were moistened with 2 mL of distilled water because they were becoming dry. Seeds that had not germinated after 127 days were returned to 20°C for 3 days until they germinated.

### Phenotypic assessment, HN types and test of the Mendelian ratios

In 2010 in Motueka and in 2011 in Angers, seedlings were classified into three groups according to the morphological appearance of HN and a chronological criterion, as observed at 1 and 3 months after germination. In 2014 in Motueka, seedlings were again classified into three groups, using the same criteria; however, a protocol for a more detailed assessment of the HN phenotype was set up. According to this protocol, the dry weight was measured for all seeds individually, as well as the weight and the radicle length of each germinated seed at the planting date. Moreover, the number of seedlings that stopped growing, were necrotic or dead, and the number of seedlings that were growing normally were counted at 30, 50 and 85 days after planting. The plant height was measured at all these assessments, and the plant condition (chlorosis, presence of necrosis and cupped leaves) was noted. At the first assessment, the leaf area was also determined, and at the final assessment, the number of buds was noted. To measure the leaf area, seedlings were placed on a white background and photographed from above; then images were processed and scaled using Adobe Lightroom5 and leaf area was calculated using ImageJ (http://imagej.nih.gov/ij/).

The segregation ratios for the seedling types were computed and compared with various Mendelian ratios (1:1, 1:3, 1:7 and 3:13), corresponding to various genetic models, using chi-square (*χ*^2^) tests.

### DNA extraction and design of high-resolution melting markers for HN

In both 2010 in Motueka and 2011 in Angers, leaves from some of the necrotic seedlings were collected for DNA extraction before they died. Genomic DNA was extracted using the QIAGEN DNeasy Plant Kit (QIAGEN GmbH, Hilden, Germany) or the NucleoSpin^®^ 96 Plant II (Macherey-Nagel GmbH & Co. KG). DNA quantifications were carried out using a NanoDrop^™^ 2000c spectrophotometer (Thermo Fisher Scientific Inc., Carlsbad, CA, USA).

In Montanari *et al*,^33^ the single-nucleotide polymorphism (SNP) and simple sequence repeat (SSR)–based parental genetic maps of PEAR3× ‘Moonglow’ were constructed using only non-necrotic seedlings, since this population was planned to be employed in quantitative trait locus (QTL) detection studies. In order to identify loci that were potentially involved in control of HN, these maps were searched for regions where the markers showed distorted segregation ratios by plotting the Minor Allele Frequency (MAF) value for each marker used for map construction against its position on the LG, where MAF lower than 0.35 indicated severe segregation distortion. Two to four SNP markers were randomly selected within each of the distorted regions on LGs 2, 5 and 10 of PEAR3 and LGs 1, 9, 10 and 16 of ‘Moonglow’, and high-resolution melting (HRM) markers were developed from these SNPs (Supplementary Table 1). Polymerase chain reaction (PCR) primers were designed around SNPs using Primer3 software^34^ (http://primer3.ut.ee/) with the following criteria: (i) PCR product size between 50 and 200 base pairs (bp); (ii) primer size between 18 and 25 bases; (iii) optimal melting temperature (Tm) of 59°C; (iv) GC content of each primer between 40% and 55%; (v) maximum alignment score and global alignment score for self-complementarity and complementarity between primer pairs set to 4 and 1, respectively. The quality of the primers was controlled by BLASTn queries against the ‘Bartlett’ v1.0 genome.^35^

Additional HRM primers were designed, with the same criteria as above, on the sequences of putative candidate lethal genes (NB-LRR genes) annotated in apple genome regions^36^ orthologous to the distorted ones on LGs 1, 5 and 10 (Supplementary Table 2).

PCRs and HRM analyses were performed on DNA from necrotic (148) and non-necrotic (105) individuals (these last ones included individuals who had been used for the genetic map construction) using a LightCycler^®^ 480 instrument (Roche Diagnostics GmbH, Roche Applied Science, Mannheim, Germany) as described by Guitton *et al*.^37^

### Genetic linkage map analysis

The new HRM markers and the genotypes of the necrotic seedlings were added to the SNP and SSR dataset described in Montanari *et al*,^33^ and updated parental genetic maps were constructed for the target LGs using JoinMap v4.0 software^38^ following the double-pseudo testcross mapping strategy.^39^ The LGs were determined with a minimum LOD score of 4 for grouping, and the Kosambi function was used for map calculation. Maps were drawn using MapChart 2.2.^40^

### SSR analysis of regions associated with HN

Eighteen microsatellite markers, selected from published apple and pear SSRs^41–45^ within the regions associated with HN (detected by the HRM marker analysis), and the SSR markers CH03a09 and CHVf1, previously mapped to LG5 of PEAR3 and LG1 of ‘Moonglow’, respectively,^33^ were used to genotype both the necrotic and non-necrotic individuals, in order to reduce the interval of the regions linked to lethality. PCRs consisted of 20 ng of genomic DNA, 1x QIAGEN Multiplex PCR Master Mix and 0.2 μM of each forward and reverse primer, in a final volume of 12.5 μL. Three to four SSRs with fluorescent-labelled primers were multiplexed and amplified using an Applied Biosystems^®^ GeneAmp^®^ PCR System 9700 (Applied Biosystems^®^ by Life Technologies™) at Fondazione Edmund Mach (Italy). Multiplex PCRs were performed as described by Teixeira and Bernasconi,^46^ with some modifications: the initial denaturation step was followed by five touchdown cycles with a decrease of 1°C/cycle, and the main amplification reactions consisted of 35 cycles. Fragments were analysed as outlined by Montanari *et al*.^33^ All the SSR markers, as well as one necrotic phenotype, were then incorporated in the PEAR3 and ‘Moonglow’ genetic maps.

In order to identify the origin of the incompatible alleles, accessions from the PEAR3 and ‘Moonglow’ pedigrees, including *P. communis* Michigan-US 437, ‘Roi Charles de Würtemberg’, ‘Williams Bon Chrétien’ and ‘Seckel’ and *P.* × *bretschneideri* ‘Xuehuali’, were screened with CHVf1, Hi04d02, CH05f06, CH02f06, Hi08g12, CN493139, CN444636 and Hi24f04 markers.

### Alignment of the regions associated with HN with other SNP-based genetic maps for pear

The regions associated with ‘Type1’ and ‘Type 2’ HN were aligned with homologous regions in the segregating pear populations ‘Old Home’ × ‘Louise Bonne Jersey’ (OH × LBJ), PEAR1 × PEAR2, POP356 and POP369, which were screened with the Illumina apple and pear Infinium^®^ II 9K SNP array,^33^ with the aim of identifying SNP markers with a strong or completely (i.e. with an entire genotypic class missing) distorted segregation, which may have been filtered out during the initial SNP array analysis in PEAR3 × ‘Moonglow’ because of the very low MAF.

## Results

### Phenotypic evaluation of HN

**Figure 1:**
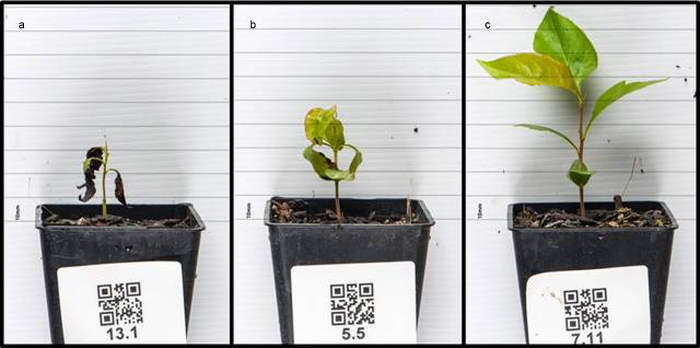
HN phenotypes in the *Pyrus* interspecific PEAR3 × ‘Moonglow’ population. Three distinct phenotypes were observed in the seedlings. Pictures were taken 30 days after germination: (a) ‘Type 1’ seedlings had stopped growing and chlorosis and necrotic lesions were apparent on their leaves; (b) ‘Type 2’ seedlings initially grew normally; however, their leaves began to cup downwards and to become chlorotic and necrotic. (c) ‘Type 3’ seedlings grew normally.

The seeds from the PEAR3 × ‘Moonglow’ cross had high rates of germination across years and locations. In total, 704 seeds out of 775, 657 out of 728 and 227 out of 240 germinated in Motueka in 2010, in Angers in 2011 and in Motueka in 2014, respectively, for an overall germination rate greater than 90%. The alternation of cold and warm temperature treatments on seeds improved germination in 2014. Three distinct phenotypic classes were identified in the segregating population over both sites and years. ‘Type 1’ seedlings ceased growing very soon after germination, and chlorosis and necrotic lesions were apparent on their leaves (Figure 1a). These seedlings died within 1 month after germination or remained less than 50 mm in height with small leaves. ‘Type 2’ seedlings initially developed normally; however, the leaves began to cup downwards and became chlorotic and necrotic (Figure 1b), with these characteristics becoming increasingly apparent by 50 and 85 days after planting. Within 3 months after germination, plant development stopped and the seedlings did not grow higher than 150 mm, progressively degenerating with time. ‘Type 3’ seedlings grew normally (Figure 1c).

**Figure 2:**
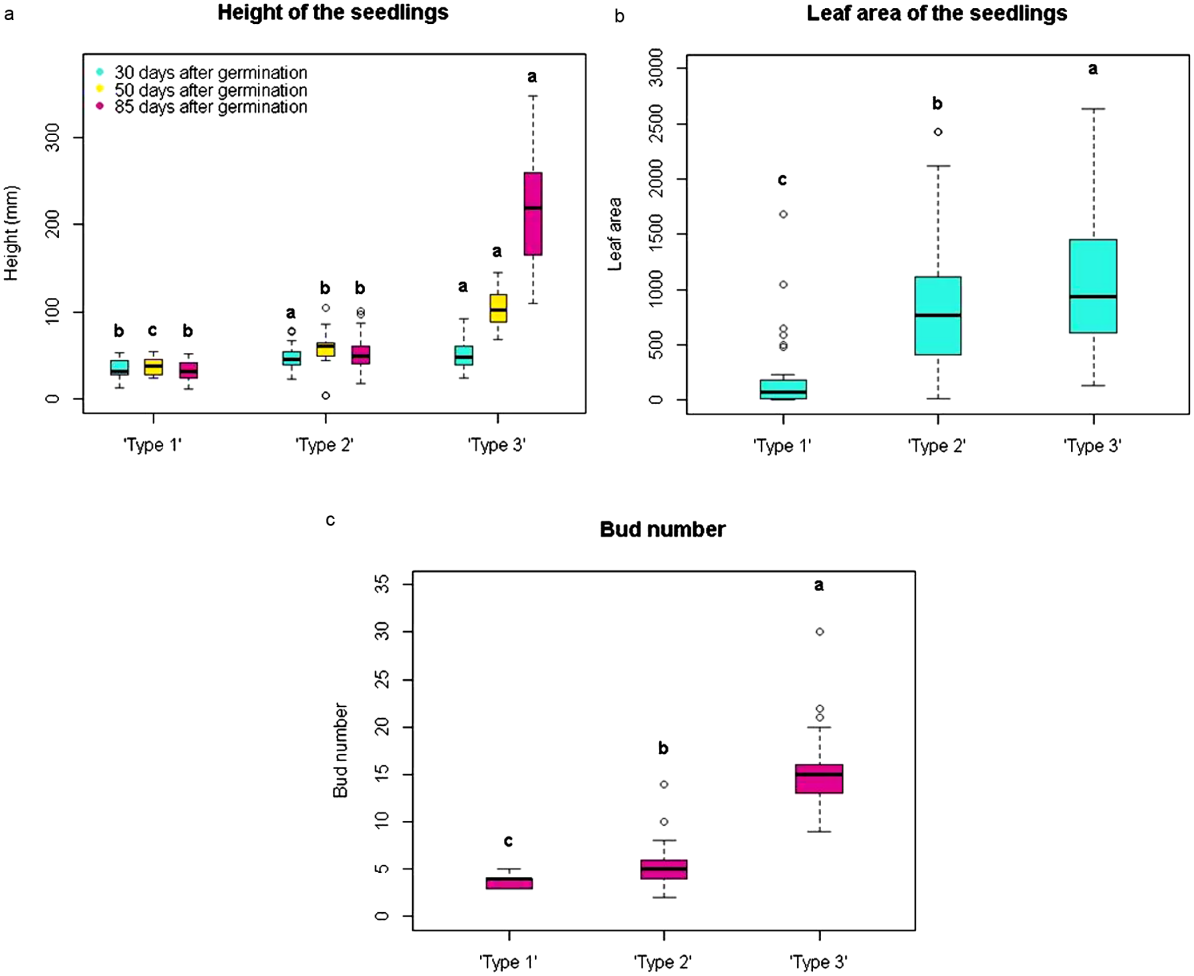
Differences in plant development amongst ‘Type 1’, ‘Type 2’ and ‘Type 3’ seedlings in the *Pyrus* PEAR3 × ‘Moonglow’ progeny sown in Motueka in 2014. The letters on top of each box (a, b and c) represent significant differences (according to the Student–Newman–Keuls test). (a) Height of the seedlings measured at 30 (in light blue), 50 (in yellow) and 85 (in purple) days after germination. Significant differences amongst the three types are shown for each assessment. (b) Leaf area measured at 30 days after germination. (c) Average number of buds counted at 85 days after germination.

In 2014 at Motueka, while seedlings were classified basing on the same criteria as in the two previous experiments (morphological and chronological appearance of HN), a detailed protocol for the evaluation of the three phenotypes was applied. At 30 days after germination, ‘Type 1’ seedlings were significantly smaller (according to Student–Newman–Keuls test) than the seedlings in the other two phenotypic classes, while there was no difference between the heights of ‘Type 2’ and ‘Type 3’ seedlings (Figure 2a). In contrast, the height at 50 and 85 days of ‘Type 3’ plants was significantly greater than that of the seedlings in the other two phenotypic classes (Figure 2a). ‘Type 2’ seedlings were taller than ‘Type 1’ seedlings at 50 days, but not at 85 days, which could be explained with the bending down of the stems due to extended necrosis. Moreover, the leaf area (measured at 30 days) (Figure 2b) and the bud number (measured at 85 days) (Figure 2c) were significantly different amongst the three classes. No significant differences were observed for the seed weight, both dry and at planting, or for the radicle length.

### Genetic model for ‘Type 1’ and ‘Type 2’ HN

The observed segregation ratios for ‘Type 1’:‘Type 2’:‘Type 3’ phenotypes in the PEAR3 × ‘Moonglow’ population were 153:271:280 (22%–38%–40%), 101:260:296 (15%–40%–45%) and 44:79:104 (19%–35%–46%) in Motueka 2010, Angers 2011 and Motueka 2014, respectively (Table 1). A *χ*^2^ test was performed in order to increase understanding of the genetic basis of the observed segregation ratios for ‘Type 1’:‘Type 2’ + ‘Type 3’ and for ‘Type 2’: ‘Type 3’. At *α* = 0.05, the progeny segregation for ‘Type1’:‘Type2’ + ‘Type 3’ in the Motueka 2014 experiment was consistent with a 1:3 (*ρ =* 0.051) or a 3:13 (*ρ =* 0.806) ratio, while the segregations observed in Motueka 2010 and in Angers 2011 experiments did not fit any of the Mendelian ratios tested. However, at α = 0.01 in Motueka 2010, they were consistent with the 1:3 (*ρ* = 0.045) and the 3:13 (*ρ* = 0.043) ratios, and in Angers 2011 with the 1:7 (*ρ* = 0.027, data not reported) and the 3:13 (*ρ* = 0.027) ratios. The pooled data were not significantly different from the 3:13 ratio (*ρ* = 0.987), as shown by the *χ*^2^ test performed on the sum of the three experiments pooled for each class (Pooled *χ*^2^) (Table 1). Consequently, both the 1:3 and the 3:13 ratios were taken into account, while the 1:7 ratio appeared more unlikely.

The observed ‘Type 2’:‘Type 3’ ratio fitted well the 1:1 Mendelian ratio at a risk of *α* = 0.05 (*ρ* = 0.699, *ρ* = 0.127 and *ρ* = 0.064, respectively for Motueka 2010, Angers 2011 and Motueka 2014), and the three experiments were rather homogeneous for these data, with a pooled dataset generating a significant ratio (*ρ* = 0.051) (Table 1).

### Detection of candidate genomic regions linked to HN

**Figure 3:**
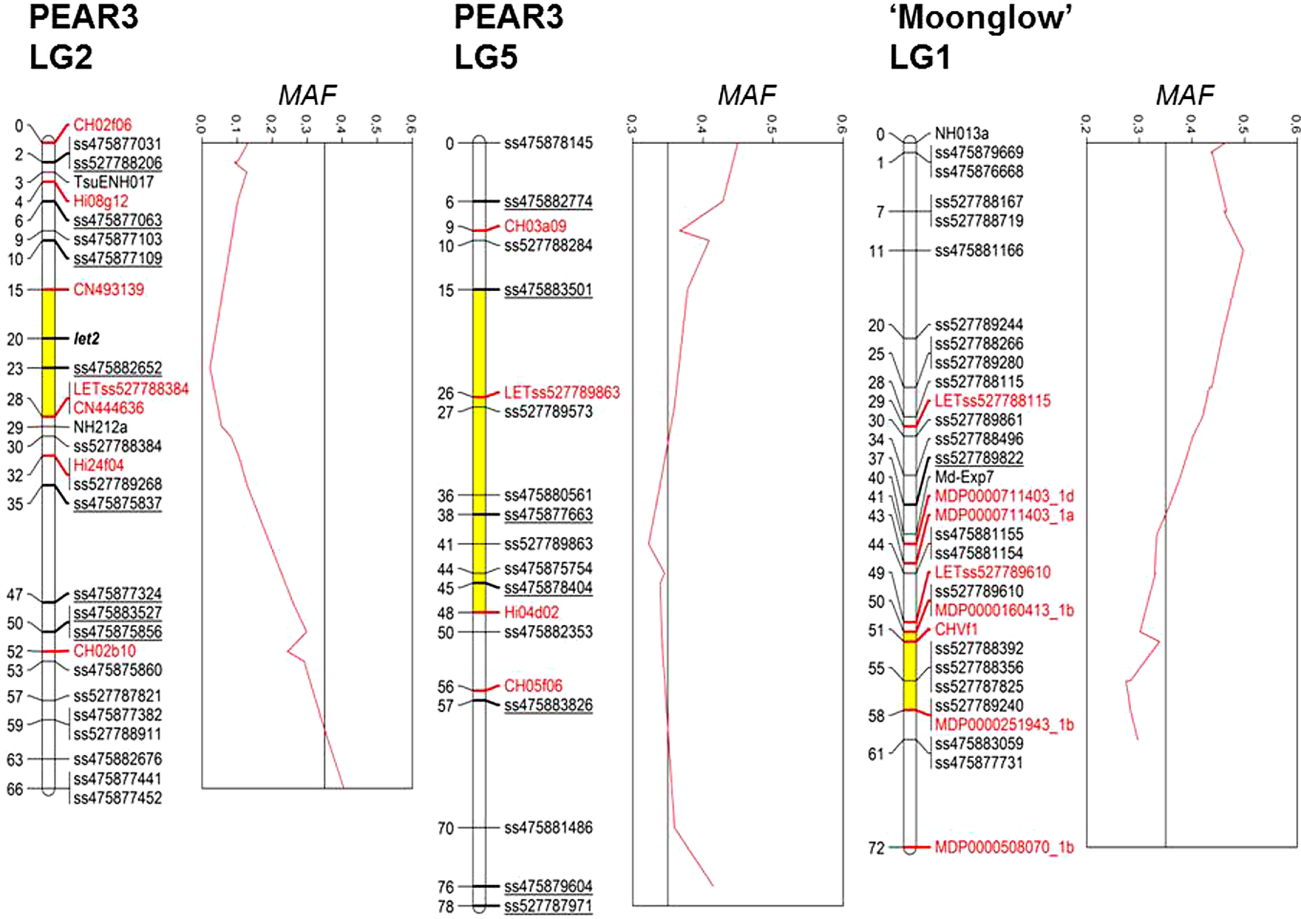
Genetic map of LG2 and LG5 of *Pyrus* PEAR3 and LG1 of ‘Moonglow’, indicating regions of segregation distortion. The MAF (red curves) is presented as a measure of segregation distortion of the markers evaluated on non-necrotic progeny. HRM and SSR markers used for ‘Type 1’ and ‘Type 2’ screening are highlighted in red. Newly mapped SNPs with respect to the map of Montanari *et al*^33^ are underlined. The regions involved in HN are marked in yellow. The locus *let2* linked to ‘Type 2’ phenotype is in bold and italic.

Segregation distortion was detected on seven LGs: 2, 5 and 10 of PEAR3 and 1, 9, 10 and 16 of ‘Moonglow’, on the basis of deviation below a MAF of 0.35 for individual markers (Supplementary Figure 2). DNA extracted from 55 ‘Type 1’ and 93 ‘Type 2’ necrotic seedlings (for a total of 148 individuals), plus 105 non-necrotic ‘Type 3’ seedlings, was screened with newly developed HRM markers designed from SNPs with distorted segregation frequency mapping to these seven candidate regions for HN. Twelve HRM markers out of 23 were polymorphic and were distributed over all the candidate regions, with 10 of them mapping close to the genetic location of the SNP marker from which they had been developed (Supplementary Table 1, Figure 3). Thirty-one new HRM markers were also developed from putative candidate lethal genes (NB-LRR genes) on LGs 1, 5 and 10, and 15 were polymorphic, with 10 mapping to the locations predicted from the apple whole-genome sequence^36^ (Supplementary Table 2, Figure 3).

A *χ*^2^ test of independence of ‘Type 1’ versus ‘Type 2’ + ‘Type 3’ individuals for the newly designed HRM markers indicated that the genotypic ratios were strongly skewed (*ρ* = 1) for the combination of markers mapping to LG5 of PEAR3 and LG1 of ‘Moonglow’. The most extreme situation was observed for markers LETss527789863 from LG5 of PEAR3 and MDP0000160413_LG1b from LG1 of ‘Moonglow’, for which 44 out of 54 ‘Type 1’ seedlings (85.5%) carried both the *b* alleles of the first marker and the *n* allele of the second marker, while only eight out of 166 ‘Type 2’ + ‘Type 3’ seedlings (4.8%) carried these alleles in combination (Table 2). This demonstrated a linkage between the ‘Type 1’ phenotype and a combination of loci mapping to PEAR3 LG5 and ‘Moonglow’ LG1.

Likewise, a *χ*^2^ test of independence of ‘Type 2’ versus ‘Type 3’ individuals indicated that LETss527788384 from LG2 of the interspecific parent PEAR3 was linked to the ‘Type 2’ phenotype (Table 2). Based on this observation, a linkage analysis was performed by considering the ‘Type 2’ HN as a phenotypic marker segregating 〈lmxll〉 (consistent with the 1:1 segregation ratio observed for ‘Type 2’:‘Type 3’). The corresponding locus, named *let2* (as the “lethal gene causing Type 2 HN”), was mapped to LG2 of PEAR3, 8cM upstream from the LETss527788384 marker (Figure 3). This same approach could not be applied to ‘Type 1’ HN, since it was not possible to map an interaction between the two genes located on different LGs.

### Refinement of the intervals of the regions linked to HN and pedigree analysis of the incompatible alleles

Microsatellite markers were used to reduce the interval of the three regions linked to HN, on LGs 2 and 5 of PEAR3 and LG1 of ‘Moonglow’. When 18 SSR markers from LGs 2 and 5 were tested on the PEAR3 × ‘Moonglow’ population, 7 and 4, respectively, were polymorphic (Supplementary Table 3). These 11 SSRs, plus 2 other SSRs previously mapped to LG5 of PEAR3 (CH03a09) and LG1 of ‘Moonglow’ (CHVf1), were used to screen DNA from 49 ‘Type1’, 76 ‘Type 2’ and 74 ‘Type 3’ seedlings. Five and two of the newly tested SSR markers mapped to the LGs 2 and 5 of PEAR3, respectively, while Ch05e06, CN581493 and Hi02a07 were homozygous in PEAR3 and mapped only to ‘Moonglow’, and CN445599 did not map (Supplementary Table 3, Figure 3).

For LG5 and LG1 markers, the frequency of the ‘Type 1’ individuals carrying the incompatible alleles was examined (Table 3). On LG5, an allele of HRM marker LETss527789863 derived from PEAR3 (denoted as *b*) had the highest frequency (90.7%) in ‘Type 1’ seedlings. Three SSRs were mapped close to this locus on LG5: CH03a09, Hi04d02 and CH05f06; however, the frequency of ‘Type 1’ seedlings bringing the incompatible allele of the SSR CH03a09 (115 bp, denoted as *l*) was only 48.8%, and hence, this marker might not belong to the region linked to ‘Type 1’ lethality. On LG1, a null allele of SSR marker CHVf1 inherited from ‘Moonglow’ (denoted as *p*) had the highest frequency (86.5%) in ‘Type 1’ seedlings. Concerning LG2 of PEAR3, alleles denoted as *e* (148 bp), *m* and *a* (243 bp) for the markers CN493139, LETss527788384 and CN444636, respectively, showed the highest frequencies (87.8%, 87.6% and 87.7%, respectively) in ‘Type 2’ seedlings (Table 3).

**Figure 4:**
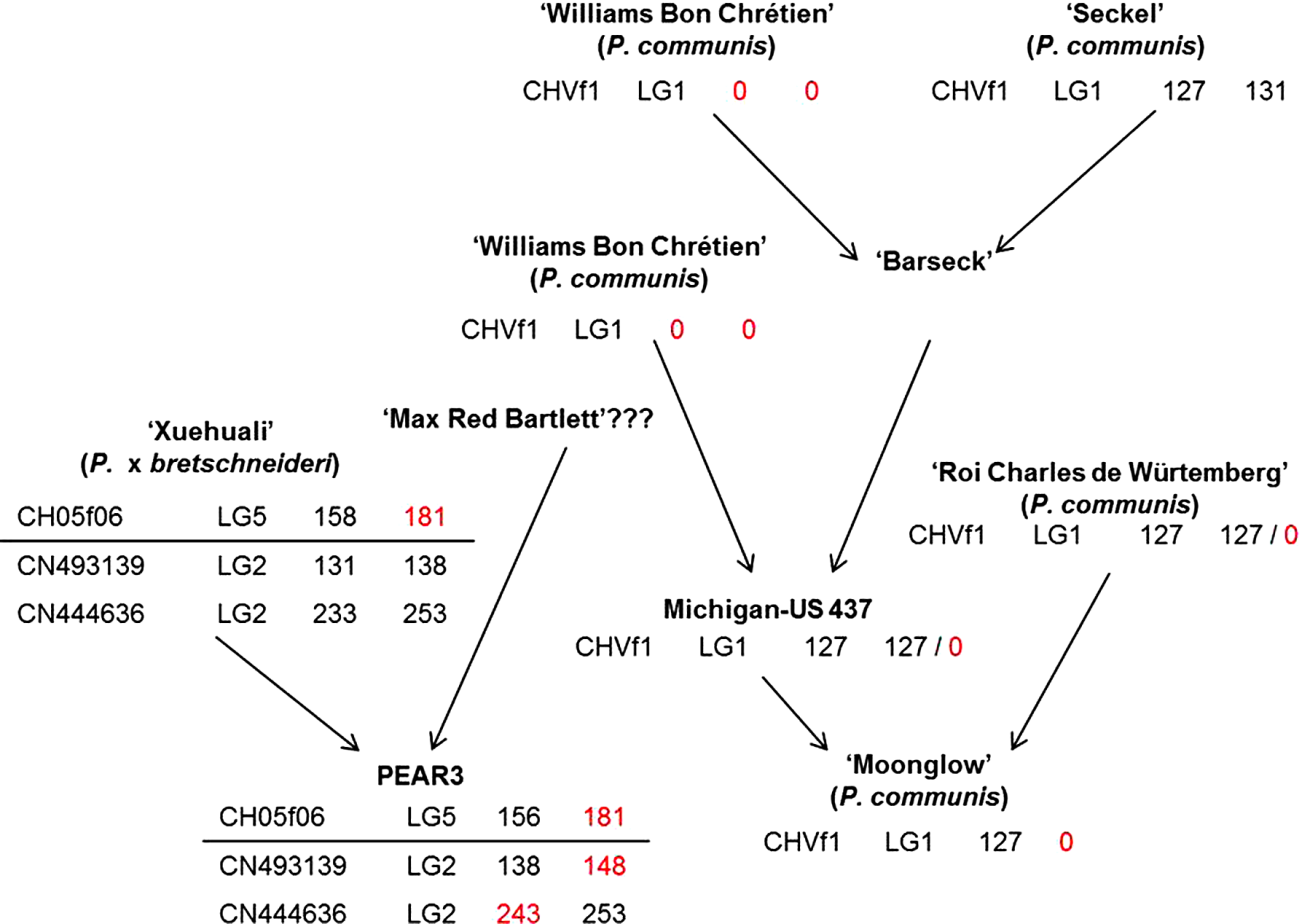
Inheritance of the lethal alleles in the *Pyrus* PEAR3 × ‘Moonglow’ pedigree. Progenitors of PEAR3 and ‘Moonglow’ were scanned with SSR markers mapped within the regions involved in HN. For each marker, the incompatible allele (in bp) is highlighted in red.

The five SSR markers located within the regions discovered to be linked to HN (Hi04d02 and CH05f06 on PEAR3 LG5; CHVf1 on ‘Moonglow’ LG1; CN493139 and CN444636 on PEAR3 LG2) were used to genotype the progenitors of PEAR3 and ‘Moonglow’, in order to identify the origin of the incompatible alleles (Supplementary Table 4). ‘Max Red Bartlett’, which is a red-skinned form of ‘Williams Bon Chrétien’, was found not to be related to PEAR3 by the SSR analysis (Supplementary Table 4, Figure 4), and the PEAR3 male parent remains unknown.

PEAR3 carries an 181 bp allele of CH05f06 associated with ‘Type 1’ HN and inherited from ‘Xuehuali’, while it was not possible to ascertain the origin of the 164 bp allele associated with incompatibility at Hi04d02, since PEAR3 exhibits both alleles carried by ‘Xuehuali’ (Supplementary Table 4, Figure 4). The parents of ‘Moonglow’, Michigan-US 437 and ‘Roi Charles de Würtemberg’ showed only one peak at 127 bp for CHVf1, and hence, they both potentially bring the null allele associated with ‘Type 1’ HN at this locus (Supplementary Table 4, Figure 4).

The ‘Type 2’ incompatibility was associated with the 148 bp and 243 bp alleles of SSR markers CN493139 and CN444636, respectively; however, ‘Xuehuali’ did not carry either of these alleles (Supplementary Table 4, Figure 4), which might thus have been inherited from the unknown male parent of PEAR3.

### Strongly distorted SNPs map to the regions associated with HN

Alignment of the three regions linked to HN with homologous regions in the pear populations OH × LBJ, PEAR1 × PEAR2, POP356 and POP369^33^ enabled us to map eight, seven and one strongly distorted SNPs to LGs 2 and 5 of PEAR3 and to LG1 of ‘Moonglow’, respectively (Supplementary Table 5, Figure 3). Moreover, five SNPs with completely distorted segregations were identified, all of which mapped to LG2 in at least one of the other pear maps and were heterozygous in PEAR3 (Table 3). The location on PEAR3 LG2 of those SNP markers could not be identified with certainty, since they cannot be mapped; however, we could ascertain that ss527787834 (segregating 〈abxaa〉 and with *ab* genotype missing amongst ‘Type 3’ individuals) was located between ss527788206 and ss527789268, in the region linked to ‘Type 2’ lethality (Figure 3).

Following the rearrangement of the markers on LG2 of PEAR3 with respect to the original map of Montanari *et al*,^33^ after the addition of the new HRM, SSR and SNP markers, the peak of distortion could be identified within the region linked to lethality, as was also exhibited for PEAR3 LG5 and ‘Moonglow’ LG1 (Figure 3).

## Discussion

The cross between the first-generation interspecific accession PEAR3 (*P.* × *bretschneideri* × *P. communis*) and the European pear ‘Moonglow’ (*P. communis*) generated a large proportion (more than 50%) of non viable seedlings, which exhibited a typical HN phenotype.^2,3^ Screening with molecular markers enabled us to identify three chromosome regions associated with this phenomenon. Segregation analysis of phenotypes showed that BDM-like incompatibilities involving epistasis amongst different loci were the basis of HN in this pear population, a finding that is consistent with reports for other plant species.^6,47,48^ Since an autoimmune response is likely to occur in incompatible combinations showing the HN phenotype,^1,10,11^ we discuss our findings in relation to previously mapped resistances in pear. Furthermore, we identified SSR markers linked to the lethal genes, which were used to perform a pedigree analysis that outlined the existence of postzygotic gene-flow barriers between the two different *Pyrus* species.

### Two independent postzygotic incompatibilities

**Figure 5:**
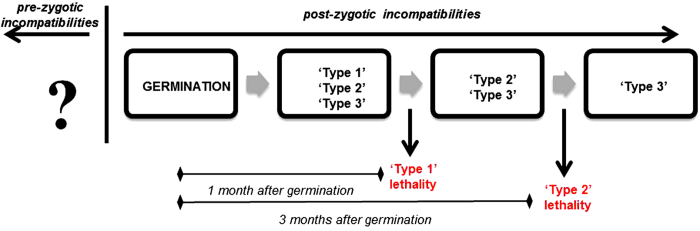
Timing of the expression of the genetic incompatibilities and lethality that occur in the *Pyrus* PEAR3 × ‘Moonglow’ population. A timeline is drawn to show when ‘Type 1’ and ‘Type 2’ seedlings die or irreversibly stop growing and necrotize.

The presence of necrotic lesions in both ‘Type 1’ and ‘Type 2’ phenotypes indicated that the lethality observed in these seedlings was due to HN. We hypothesized that ‘Type 1’ and ‘Type 2’ lethality had independent biological and genetic causes. The incompatibility causing the ‘Type 1’ plants to become stunted and die acted quickly, causing the lethality of the plantlets within 30 days after germination, while the ‘Type 2’ dwarfism acted more slowly, reaching its complete expression only 3 months after germination. Figure 5 presents a model for pre- and postzygotic hybrid lethality, showing at which stage the ‘Type 1’ and ‘Type 2’ phenomena are fully expressed. The existence of a number of highly distorted regions in the parental genetic maps (Supplementary Figure 2) suggested the presence of both prezygotic (not characterized) and postzygotic lethal loci affecting the offspring development. Lethal genes involved in ‘Type 1’ and ‘Type 2’ HN were likely to be located in some of these regions.

The different timing of the expression of ‘Type 1’ and ‘Type 2’ lethality indicated that they were caused by two independent postzygotic incompatibilities. This hypothesis was supported by the genetic markers analysis, which clearly showed these two phenotypes to be due to different and unlinked loci (Table 2).

### Negative epistatic interactions cause ‘Type 1’ and ‘Type 2’ lethality

The segregation ratios for the ‘Type 1’:‘Type 2’ + ‘Type 3’ were heterogeneous amongst Motueka 2010, Angers 2011 and Motueka 2014 seedlings. They appeared to fit a 1:3 or a 3:13 Mendelian ratio, although the *χ*^2^ ρ value was above the risk of 0.05 only at the last experiment, and just below it for Motueka 2010 seedlings, while the Angers population showed discrepancy from these Mendelian ratios (Table 1). This might be attributed to a more accurate classification of the three phenotypes at Motueka in 2014, when the phenotypic assessment was more detailed than those in the two earlier experiments, and to differing environmental conditions between the two sites (Motueka and Angers), including the treatments to which seeds were subjected, with a higher number of ‘Type 1’-like seedlings in Motueka than in Angers (within the same set of seeds collected in 2010) (Table 1). Nevertheless, in all three experiments, the timing of expression of ‘Type 1’ and ‘Type 2’ lethality was the same. The 1:3 ratio indicates a recessive genetic control or the action of two loci, with several possible combinations of recessive and dominant alleles or no dominance epistasis, while the 3:13 ratio indicates a two-locus control with a recessive + dominant alleles interaction (Supplementary Table 6). The genotyping we performed on both necrotic and non-necrotic seedlings showed ‘Type 1’ lethality to be linked to two loci, one mapping to LG5 of PEAR3 and one to LG1 of ‘Moonglow’, with one quarter of all the possible genotypic combinations at these two loci being mainly represented by ‘Type 1’ seedlings (Table 2). Consequently, ‘Type 1’ versus ‘Type 2’ + ‘Type 3’ fitted a ratio of 1:3, as per the model of epistatic interaction between two loci with no dominance (Supplementary Table 6), consistent with the BDM model of hybrid incompatibility.^4^

The 1:1 ratio of the ‘Type 2’ phenotype with normally growing ‘Type 3’ seedlings (Table 1), homogenous across the three experiments, indicated a single-locus control, with a no-dominance epistasis between two different lethal alleles, each derived from a different parent, or a two-locus control involving one recessive and one dominant allele, or two dominant alleles (Supplementary Table 6). Only markers mapping to LG2 of PEAR3 were found to be associated with ‘Type 2’ lethality (Table 2). However, Supplementary Figure 3 illustrates that it is also possible that the LG2 locus interacts with another, yet unmapped, locus that would be homozygous for the viable allele in PEAR3 (*aa*) and homozygous for the lethal allele in ‘Moonglow’ (*ll*). In this case, all progenies would have genotype *al* and contribute the lethal allele, but the ‘Type 2’ inviability would be expressed only in the simultaneous presence of the lethal allele of the gene on LG2. Since no segregation distortion was visible for this second locus in the F1 progeny, its chromosomal location could not be identified. This two-locus hypothesis is more probable than the single-locus one, because postzygotic incompatibilities have usually been demonstrated to be caused by epistatic interactions between at least two genes.^2,4^ Backcrossing the viable F1 progeny (which carries the lethal allele only at the unknown locus and not at the LG2 locus) with PEAR3 would validate this hypothesis (Supplementary Figure 3).

Additional HRM markers may be designed on the completely distorted SNPs mapped to LG2 in the other pear segregating populations, and in particular around ss527787834, and then run on the ‘Type 2’ seedlings. Indeed, markers closely linked to the lethal gene should have a genotypic ratio of 1:0 for the incompatible:compatible genotype.

### Resistance genes might be involved in ‘Type 1’ and ‘Type 2’ inviability

The frequency of ‘Type 1’ seedlings carrying the incompatible allele inherited from LG1 of ‘Moonglow’ is higher for SSR CHVf1 than for the markers flanking it (Table 3), indicating that the lethal gene is closely linked to this SSR and located between markers MDP0000160413_LG1b and MDP0000251943_LG1b, which spanned a region of 8 cM (Figure 3). In apple, SSR CHVf1is tightly linked to two major genes conferring scab (*Venturia inaequalis*) resistance, *Rvi6* and *Rvi17*,^23^ historically known as *Vf*^49^ and *Va1*,^50^ respectively. As the apple and pear genomes are highly syntenic,^51–53^ it is possible that a locus orthologous to the apple *Rvi6* gene is involved in ‘Type 1’ lethality in the PEAR3 × ‘Moonglow’ population. In the Japanese pear (*Pyrus pyrifolia*), the scab (*V. nashicola*) resistance gene *Vnk*, later re-named *Rvn1*, has also been mapped to LG1, although it appears to be located upstream to CHVf1, and then to the orthologous apple region carrying the *Rvi6* gene.^28,54,55^ *Rvi6* has been frequently associated with segregation distortion and HN events in apple.^24,25^ As this resistance originated from *M. floribunda*, widely used by apple breeders in interspecific crosses in order to obtain high-value cultivars with pyramided scab resistance,^56^ interspecies incompatibilities may well be at the basis of the HN in apple, as reported here for pear. It is of interest that one of two parental genetic maps constructed in a different pear interspecific population (PEAR1 × PEAR2) completely lacked LG1,^57^ which might have been caused by high segregation distortions for the markers that had been predicted from prior knowledge in pear and apple to map to the LG1.

In PEAR3 LG5, the locus interacting with the ‘Moonglow’ LG1 locus, the marker with highest frequency in ‘Type 1’ seedlings was the HRM marker LETss527789863, while the frequency decreased at SSR marker Hi04d02, and then at CH05f06 (Table 3). Moreover, the segregation distortion increased while moving down the LG from this point and increased again after SSR Hi04d02 (Figure 3). Therefore, we concluded that the lethal gene on PEAR3 LG5 might be located between LETss527789863 and Hi04d02, within a region of 22 cM. *R* genes located in this region might also be involved in ‘Type 1’ HN. Indeed, LG5 is one of the chromosomes in the *P.* × *bretschneideri* genome with the highest number of *R* paralog gene clusters,^58^ and QTLs for the resistance to *V. pirina*^57^ and *Cacopsylla pyri*^59^ were mapped to this LG in pear. Furthermore, Calenge *et al*^60^ mapped a QTL for scab resistance to LG5 in apple.

For the second class of HN, the highest frequencies of ‘Type 2’ seedlings bringing the incompatible allele were detected for markers CN493139, LETss527788384 and CN444636, mapping to LG2 of PEAR3 (Table 3), and the segregation distortion was stronger in the region within those markers. Thus, the lethal locus might be located between CN493139 and LETss527788384/CN444636 (which are almost co-mapping), within a region of 13 cM (Figure 3). Moreover, we mapped the *let2* locus, which controls the ‘Type 2’ phenotype, 5 cM downstream of CN493139 (Figure 3). In the *P.* × *bretschneideri* genome, LG2, like LG5, is rich in *R* paralog gene clusters,^58^ and several QTLs and major genes for resistances to pests and diseases in pear have been mapped to this LG.^54,57,61–64^ In particular, QTLs for the resistance to *V. pirina*^57^ and fire blight^62^ seem to co-locate with the region linked to HN. An example of epistasis between an *R* gene on LG2 and another LG causing segregation distortion is found in *Malus* × *domestica*, where the interaction between apple scab resistance loci *Rvi2* on LG2 and *Rvi6* on LG1 (formerly *Vh2* and *Vf*), first reported in Bus *et al*,^65^ has been observed frequently since then as an outcome of pyramiding these resistances in breeding programmes (Bus, VGM, unpubl. data). Hence, *R* genes might also be associated with ‘Type 2’ lethality in pear, as postulated for ‘Type 1’.

Further work is needed to test all these hypotheses. The HRM markers designed on NB-LRR genes annotated in the apple genome on LGs 1 and 5 did not map within the regions associated with HN (on LG1 of ‘Moonglow’, they were at the border of the region, while none of them mapped to LG5 of PEAR3); hence, none of those genes is a good candidate lethal gene. However, this approach could be applied on the reduced interval of the three regions for HN, on LGs 1, 2 and 5, also exploiting the information from the Chinese and the European pear genomes.^35,58^ Furthermore, increasing the greenhouse temperature might help to recover ‘Type 1’ and ‘Type 2’ seedlings, as high temperature treatments have often been reported to enable longer survival of plants with lethal forms of HN.^6,31,66,67^ This could therefore allow us to study their responses to pests and pathogens and then verify the hypothesis of the involvement of *R* genes in the incompatibilities.

### Incompatible alleles were inherited from different *Pyrus* spp.

On LG5 of PEAR3, the SSR marker CH05f06 provided sufficient information to conclude that the ‘Type 1’ incompatible allele originated from the Asian pear ‘Xuehuali’ (Supplementary Table 4, Figure 4). However, it was not possible to determine the origin of the incompatibility for the interacting locus mapped to LG1 of ‘Moonglow’, as either parent of ‘Moonglow’ (European pears Michigan-US 437 and ‘Roi Charles de Würtemberg’) could have potentially contributed the CHVf1 null allele. Nevertheless, we can still conclude that ‘Type 1’ HN resulted from the interaction between an Asian pear allele from a locus on LG5 and a European pear allele from a locus on LG1. Consequently, ‘Type 1’ HN is a typical result of interspecies gene-flow barriers, and the mutation that caused the evolution of the incompatible alleles might date back to the time when *P.* × *bretschneideri* and *P. communis* diverged.

In contrast, the ‘Type 2’ lethal allele at the locus mapped to LG2 was not derived from ‘Xuehuali’ and thus might be inherited from the unknown male parent of PEAR3 (Supplementary Table 4, Figure 4). We suggest that this LG2 allele has to interact with one from another gene inherited from ‘Moonglow’, whose position is unknown, in order to produce incompatibility (Supplementary Figure 3).

It is noteworthy that Yamamoto *et al*^68^ reported severe segregation distortion in both LGs 2 and 5 in the European pear ‘La France’ in a cross with a *P. pyrifolia* (Japanese pear) accession: lethal genes causing interspecies incompatibility might be at the basis of this segregation distortion, as in our population, although we observed the segregation distortion in the Asian cultivar (*P.* × *bretschneideri*), rather than in the European one. The species *P.* × *bretschneideri* is thought to be an interspecific hybrid of *Pyrus ussuriensis* × *Pyrus betulaefolia*; however, it may involve *P. pyrifolia.*^69^

### Additional lethal loci might be involved in other types of incompatibilities in the PEAR3 × ‘Moonglow’ population

Apart from the genomic segments identified on LGs 2 and 5 of PEAR3 and LG1 of ‘Moonglow’, distorted regions were detected on LG10 of both parents and on LGs 9 and 16 of ‘Moonglow’ (Supplementary Figure 2). However, these were not involved in either ‘Type 1’ or ‘Type 2’ lethality, since the genotypes for markers mapped to these regions were in equilibrium for both necrotic and non-necrotic seedlings (according to the *χ*^2^ test of independence). The high germination rates observed in the three experiments indicate absence of incompatibility at this stage of plant development. However, seeds were subjected to special treatments to promote germination in our study, while under natural conditions a higher number might fail to germinate. Our data did not enable us to determine whether those regions were involved in prezygotic incompatibility, or in aberrations of the germination process. Amongst the LGs exhibiting distortion, LG10 is of particular interest, not only because it is distorted in both parents but also because of the homology demonstrated between LGs 10 and 5 in both pear^58^ and apple^36^ genomes. Distorted segregations of markers mapping to LG10 have been previously reported in several apple populations.^27,70–72^

In summary, this is the first reported description of HN in *Pyrus*. We have shown that, although interspecific hybridization within this genus is possible, there are genetic barriers that might cause the loss of at least a proportion of the hybrid offspring.

Our detection of chromosome regions involved in postzygotic incompatibilities in pear hybrids is of considerable value, contributing both to studies on speciation and evolution and to breeding. Firstly, incompatibilities between two species might have arisen when they diverged in the evolutionary process, and their identification could assist in discovery of the selective events that drove the species differentiation. In particular, BDM incompatibilities, which involve allele mutations that do not lower fitness within the diverging lineages, can accumulate rapidly,^73^ and their identification might help to locate the speciation forces in the timeline.^74^ Secondly, breeders pyramiding resistances to enhance durability should note that they may end up with the loss of the desired resistance combination, because of incompatibilities skewing segregation in the progeny. In addition, genes associated with other desired traits could co-segregate with lethal genes and be lost to the breeding population. If these lethal genes appear to be conserved across different pear species, our identification of molecular markers linked to them will be useful for pear breeders, who would be able to select parents that avoid incompatible combinations potentially affecting the expression of the traits of interest.

The recent publication of the Chinese^58^ and European^35^ pear genome sequences offers the opportunity to develop new markers that can be used to further reduce the interval of the three regions linked to HN and to identify candidate lethal genes.

## Author contributions

SM, DC and LB designed the experiments. SM performed the marker development, the genotyping and the genetic mapping, and wrote the manuscript. LB performed the phenotyping experiments in NZ and PG in France. RL took the photographs used in this article and calculated the leaf area in Motueka in 2014. DC co-wrote the manuscript together with SM. LB and VGMB developed the PEAR3 × ‘Moonglow’ population. DC, LP, SEG and CED oversaw the genotyping and mapping part of the work. CED, RV, SEG and DC were the co-principal investigators on the SM PhD project that led to this study. They conceived the study and participated in its design and coordination, together with VGMB, LP and MM. All authors read and approved the final manuscript.

## Acknowledgements

SM was funded by the Fondazione Edmund Mach PhD School. We thank Chris Morgan at The New Zealand Institute for Plant & Food Research Limited for helping to design the germination protocol in 2014 and for his assistance with the pollination and the seed and plant measurements. We are also grateful to the technicians of the INRA greenhouse facilities, especially Nicolas Dousset and Michel Boucourt, for taking care of the seedling growth in 2010, and to Hélène Muranty for her help with the statistical analysis. We finally thank the Sequencing and Genotyping Platform at Fondazione Edmund Mach for running the capillary electrophoresis.

## Figures and Tables

**Table 1:** Observed phenotypic segregation ratios for HN in the *Pyrus* PEAR3 × ‘Moonglow’ population.

Location and year of experiment	Number of seedlings					1:3 segregation ratio			3:13 segregation ratio		
	‘Type 1’		‘Type 2’ + ‘Type 3’		Total	*X*^2^	*df*	*ρ*	*X*^2^	*df*	*ρ*
Motueka 2010	153	21.7%	551	78.3%	704	4.01	1	0.045	4.11	1	0.043
Angers 2011	101	15.4%	556	84.6%	657	32.48	1	1.2 *e*^-08^	4.92	1	0.027
Motueka 2014	44	19.4%	183	80.6%	227	3.82	1	0.051	0.06	1	0.806
Pooled	298	18.8%	1290	81.2%	1588	32.92	1	0.000	0.00	1	0.987

Location and year of experiment	Number of seedlings					1:1 segregation ratio		
		‘Type 2’		‘Type 3’		Total	*X*^2^	*df*	*ρ*
Motueka 2010	271	49.2%	280	50.8%	551	0.15	1	0.699
Angers 2011	260	46.8%	296	53.2%	556	2.33	1	0.127
Motueka 2014	79	43.2%	104	56.8%	183	3.42	1	0.064
Pooled	610	47.3%	680	52.7%	1290	3.80	1	0.051

For each of the three experiments (Motueka 2010, Angers 2011, and Motueka 2014), seedlings were assigned to a class (‘Type 1’, ‘Type 2’ and ‘Type 3’). The chi-square (*X*^2^) test was performed for ‘Type 1’:‘Type 2’ + ‘Type 3’ = 1:3 or 3:13 and for ‘Type 2’:‘Type 3’ = 1:1 for all three experiments individually. The Pooled *X*^2^was also calculated. The degrees of freedom (*df*) and the *ρ* values are shown. At *ρ* <0.05, the observed segregation ratios are significantly different from the expected ratios.

**Table 2:** Segregation ratios for the HRM markers mapped to the regions involved in HN in the *Pyrus* PEAR3 × ‘Moonglow’ population.

LG5 PEAR3 + LG1 ‘Moonglow’			
LETss527789863 (〈abxcd〉) + MDP0000160413_LG1b (〈nnxnp〉)			
Genotype	‘Type 1’	‘Type 2’ + ‘Type 3’	Total row
a + n	2	44	46
b + n	44	8	52
a + p	3	46	49
b + p	5	68	73
Total column	54	166	220
		*X*^2^	135.03
		*df*	7
		*ρ*	1

LG2 PEAR3			
LETss527788384 (〈lmxll〉)			
Genotype	‘Type 2’	‘Type 3’	Total row
l	11	72	83
m	78	6	84
Total column	89	78	167
		*X*^2^	106.29
		*df*	1
		*ρ*	1

The segregation ratios of the combined genotypic classes for the markers on PEAR3 LG5 and on ‘Moonglow’ LG1 are compared between ‘Type 1’ and ‘Type 2’ + ‘Type 3’ progeny. The segregation ratios of the genotypic classes for the marker on PEAR3 LG2 are compared between ‘Type 2’ and ‘Type 3’ progeny. The results of the *X*^2^ test, the degrees of freedom (*df*) and the *ρ* values are shown. At *ρ* > 0.05, the observed segregation ratios are significantly distorted. The incompatible genotypes are underlined.

**Table 3:** Proximity of the lethal genes to markers located within the regions linked to HN in the *Pyrus* PEAR3 × ‘Moonglow’ population.

**LG5 PEAR3**				
Marker	CH03a09	**LETss527789863**	Hi04d02	CH05f06
Position (cM)	9.2	26.2	48.3	56.2
Segregation	〈lmxll〉 (115:117 × 115:115)	〈abxcd〉	〈abxcd〉 (164:173 × 158:197)	〈abxcd〉 (156:181 × 173:179)
Phase	{1−}	{01}	{11}	{00}
Incompatible allele	l (115 bp)	b	a (164 bp)	b (181 bp)
% of ‘Type 1’ bringing the incompatible allele	48.8	90.7	83.7	81.3

**LG1 ‘Moonglow’**								
Marker	LETss527788115	MDP0000711403_LG1d	MDP0000711403_LG1a	LETss527789610	MDP0000160413_LG1b	**CHVf1**	MDP0000251943_LG1b	MDP0000508070_LG1b
Position (cM)	28.9	40.8	42.5	49.1	50.3	51.0	58.4	71.9
Segregation	〈nnxnp〉	〈nnxnp〉	〈nnxnp〉	〈abxcd〉	〈nnxnp〉	〈nnxnp〉 (129:129 × 127:0)	〈abxcd〉	〈nnxnp〉
Phase	{−1}	{−0}	{−1}	{10}	{−1}	{−0}	{10}	{−0}
Incompatible allele	n	p	n	d	n	p (0)	d	p
% of ‘Type 1’ bringing the incompatible allele	76.9	81.8	83.6	85.2	85.2	86.5	77.8	47.2

**LG2 PEAR3**						
Marker	CH02f06	Hi08g12	**CN493139**	**LETss527788384**	**CN444636**	Hi24f04
Position (cM)	0.0	4.5	14.6	27.7	28.2	31.7
Segregation	〈abxcd〉 (150:0 × 174:177)	〈efxeg〉 (179:196 × 179:205)	〈efxeg〉 (138:148 × 135:148)	〈lmxll〉	〈abxcd〉 (243:253 × 237:245)	〈lmxll〉 (129:139 × 144:144)
Phase	{01}	{01}	{11}	{0−}	{11}	{0−}
Incompatible allele	b (0)	f (196 bp)	e (148 bp)	m	a (243 bp)	m (139 bp)
% of ‘Type 2’ bringing the incompatible allele	70.8	84.7	87.8	87.6	87.7	83.6

For the combined loci from LG5 of PEAR3 and LG1 of ‘Moonglow’, the percentage of ‘Type 1’ contributing the incompatible alleles over the total ‘Type 1’ seedlings genotyped was calculated. For LG2 of PEAR3, the percentage of ‘Type 2’ contributing the incompatible alleles over the total ‘Type 2’ seedlings genotyped was calculated. The higher the percentage, the closer the marker is to the lethal gene. For each marker, the location on the genetic map, the allelic composition, linkage phase (with respect to the parent where the marker was mapped) and the incompatible allele are shown. The closest marker to the lethal gene is indicated in bold.
