# Supplementary material for: Genome mapping of postzygotic hybrid necrosis in an interspecific pear population
Source: Hortic Res. 2016 Jan 6;3:15064–. doi: 10.1038/hortres.2015.64 (PMC4702180; doi:10.1038/hortres.2015.64)
Supplement: Supplementary Figures S1–S3 [file hortres201564-s1.pdf]

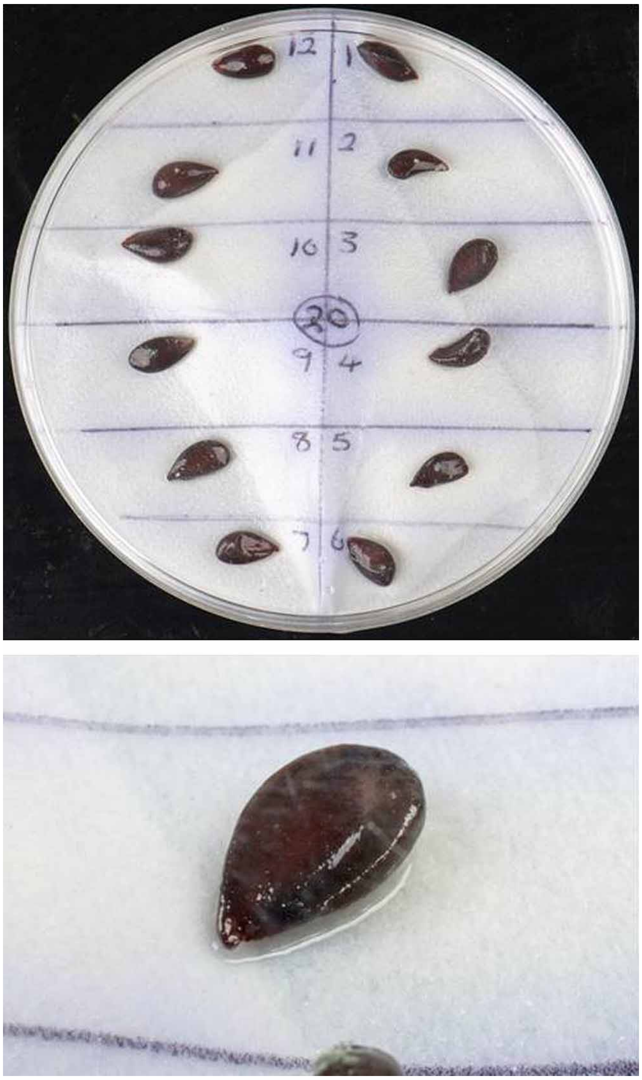

Supplementary Figure 1 Seeds from the *Pyrus* PEAR3 × 'Moonglow' cross in Motueka in 2014 subjected to treatment to enhance germination. Before sowing, seeds were dipped into a fungicide solution and then left on moist filter paper in Petri dishes until germination.

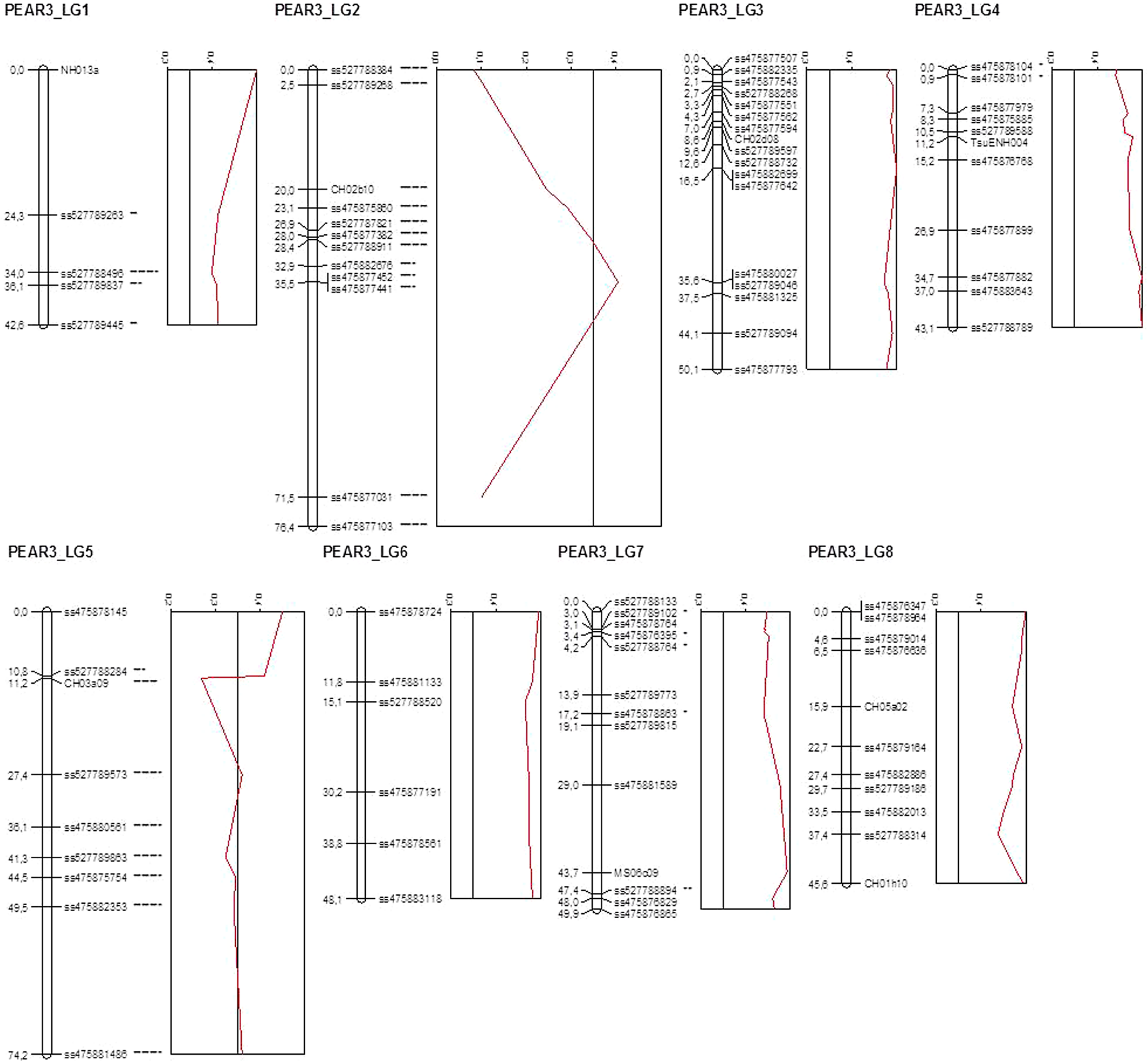

Supplementary Figure 2a Single Nucleotide Polymorphism (SNP) and microsatellite-based genetic map of PEAR3 and 'Moonglow'(33). The segregation distortion is represented by the Minor Allele Frequency (MAF) values for each marker plotted against their position in the map (red curve). The marker segregation was considered severely distorted for MAF < 0.35 (black line).

© 2015 Nanjing Agricultural University

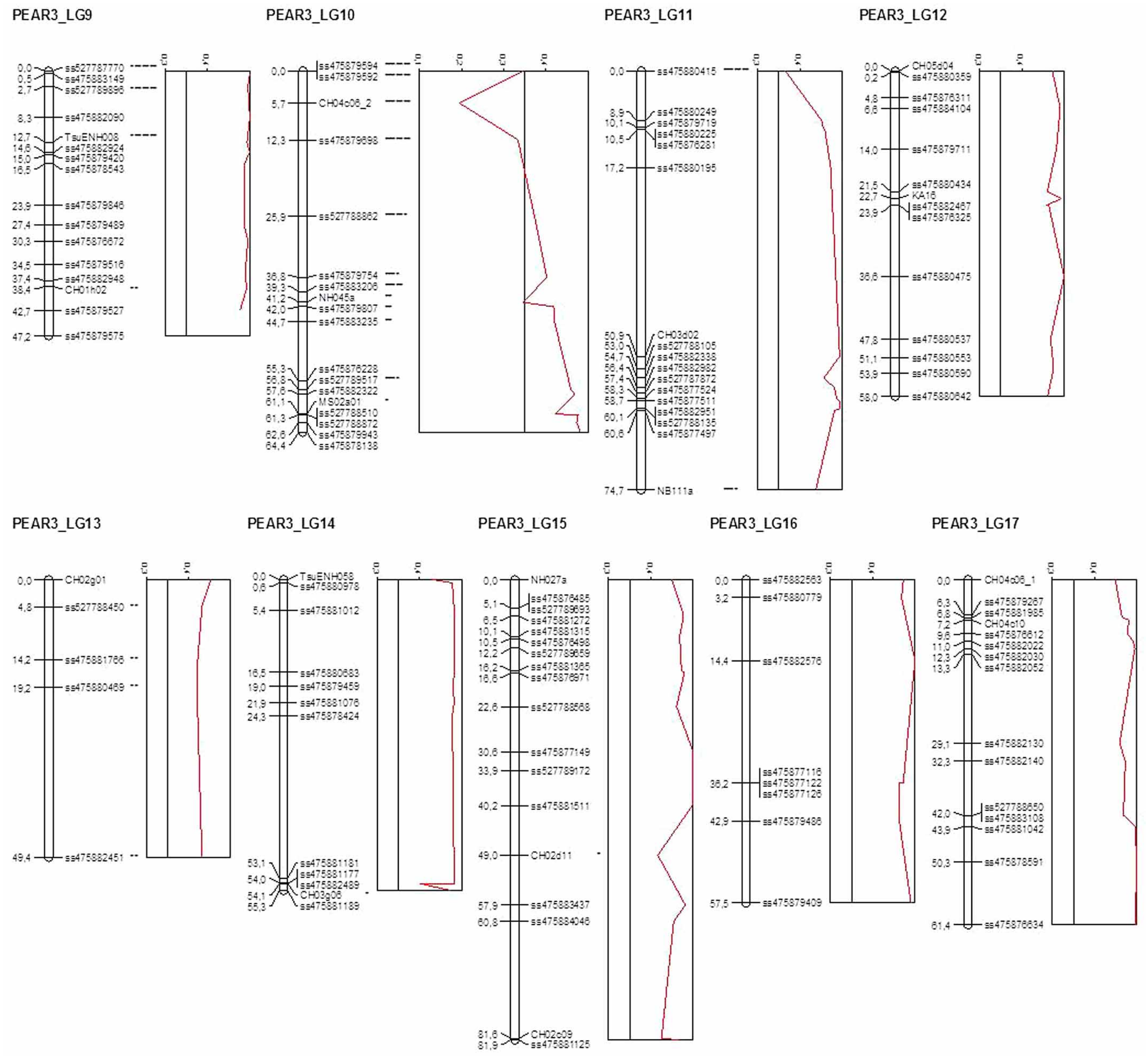

Supplementary Figure 2b Single Nucleotide Polymorphism (SNP) and microsatellite-based genetic map of PEAR3 and 'Moonglow'(33). The segregation distortion is represented by the Minor Allele Frequency (MAF) values for each marker plotted against their position in the map (red curve). The marker segregation was considered severely distorted for MAF < 0.35 (black line).

© 2015 Nanjing Agricultural University

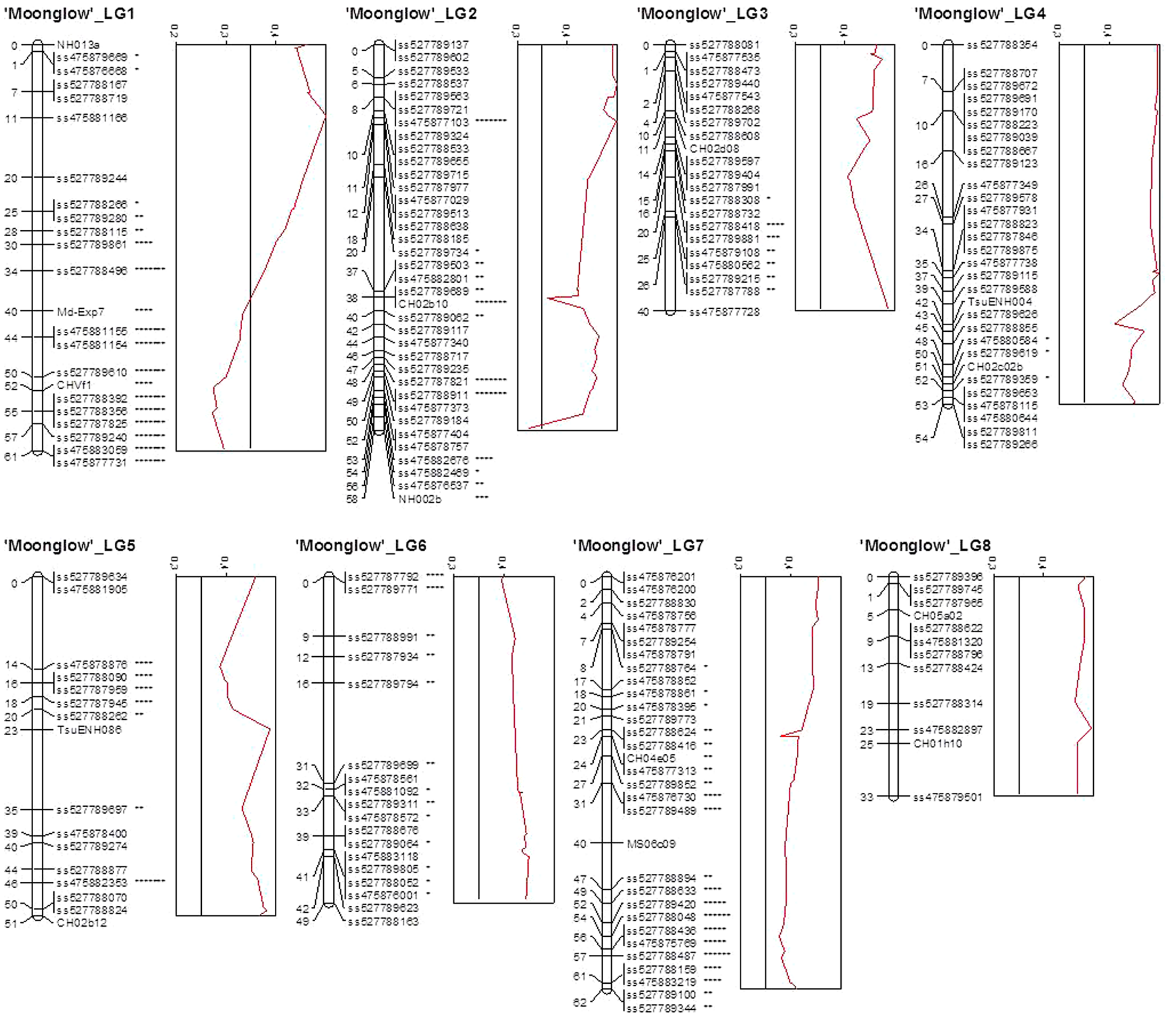

Supplementary Figure 2c Single Nucleotide Polymorphism (SNP) and microsatellite-based genetic map of PEAR3 and 'Moonglow'(33). The segregation distortion is represented by the Minor Allele Frequency (MAF) values for each marker plotted against their position in the map (red curve). The marker segregation was considered severely distorted for MAF < 0.35 (black line).

© 2015 Nanjing Agricultural University

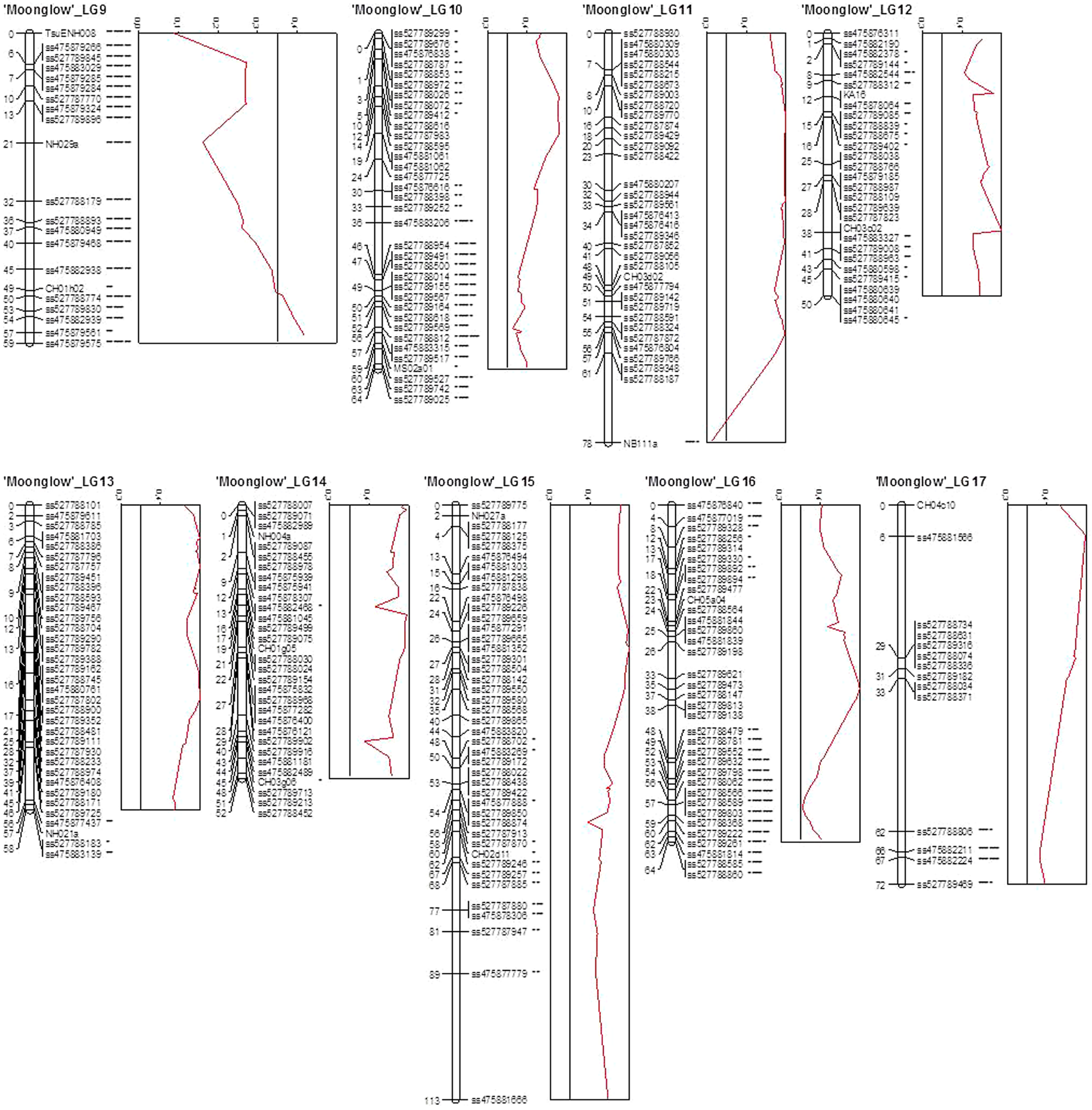

Supplementary Figure 2d Single Nucleotide Polymorphism (SNP) and microsatellite-based genetic map of PEAR3 and 'Moonglow'(33). The segregation distortion is represented by the Minor Allele Frequency (MAF) values for each marker plotted against their position in the map (red curve). The marker segregation was considered severely distorted for MAF < 0.35 (black line).

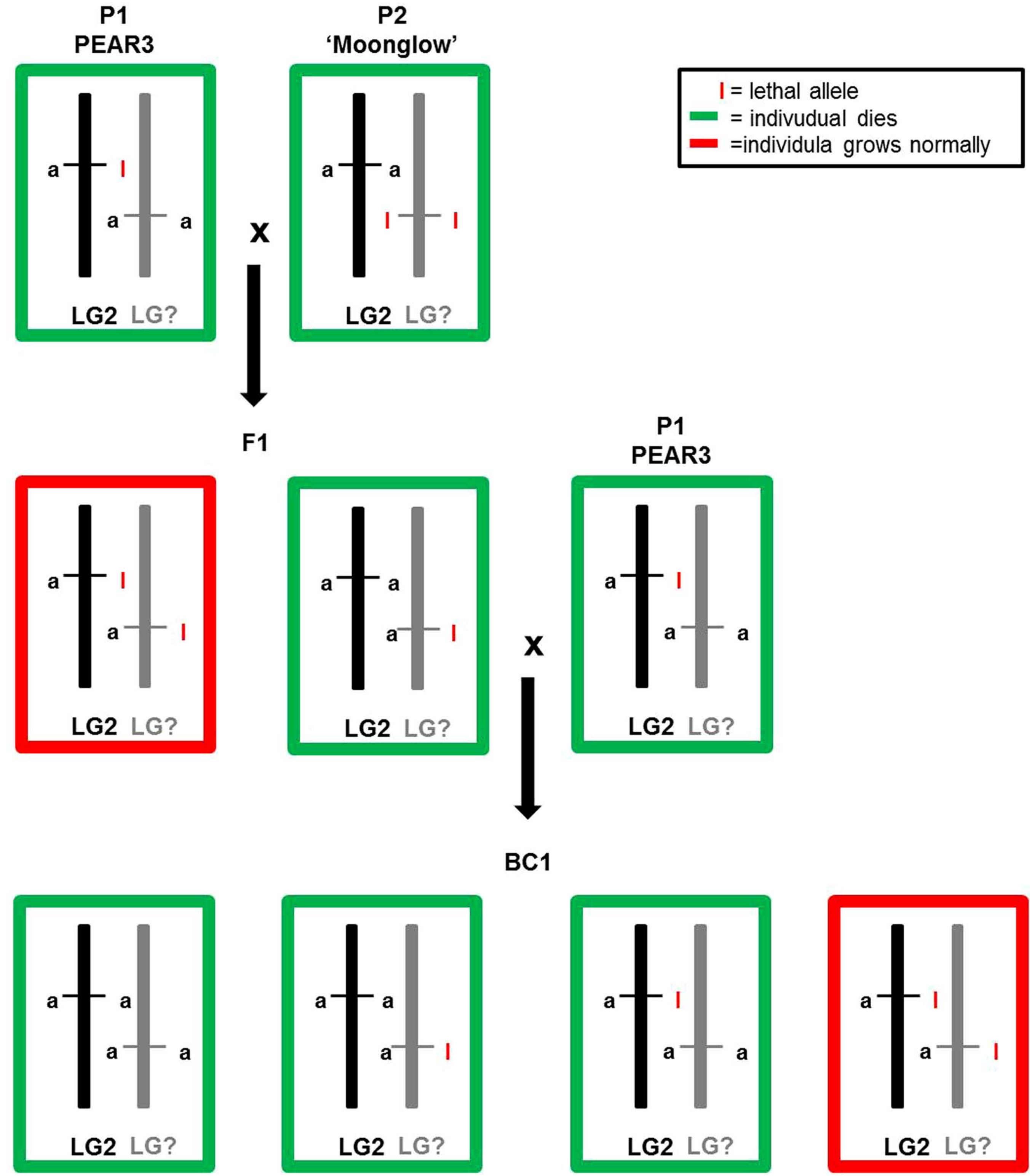

Supplementary Figure 3 Putative genetic model for the two-locus interaction causing 'Type 2' lethality in the PEAR3 × 'Moonglow' population. The individual dies only when the lethal alleles at both loci co-exist, otherwise it grows normally. Segregation distortioncan be observed in the LG2 locus, and in the BC1 for the other unknown locus.

© 2015 Nanjing Agricultural University
